# Identification of the *SAUR* Gene Family in *Pinus massoniana* and Analysis of Its Expression Patterns Under Drought Stress

**DOI:** 10.3390/biology15120962

**Published:** 2026-06-19

**Authors:** Manli Yang, Shuo Sun, Wenjuan Su, Yuke Ma, Xin Hu, Kongshu Ji

**Affiliations:** 1 State Key Laboratory of Tree Genetics and Breeding, Nanjing Forestry University, Nanjing 210037, China; 17856000818@163.com (M.Y.); 1184089140@njfu.edu.cn (S.S.); suwenj0126@163.com (W.S.); mayuke2001@163.com (Y.M.); hxneversaynever@163.com (X.H.); 2 Co-Innovation Center for Sustainable Forestry in Southern China, Nanjing Forestry University, Nanjing 210037, China

**Keywords:** *P. massoniana*, *SAUR* gene family, drought stress, auxin, expression pattern

## Abstract

*P. massoniana* is an important native economic and ecological tree species in southern China, where seasonal drought has emerged as a critical factor limiting its productivity. The *SAUR* gene family, recognized as core early auxin-responsive genes, plays a crucial role in balancing plant growth, development, and stress adaptation; however, research related to this family in conifers remains limited. Utilizing the chromosome-level genome of *P. massoniana*, this study identified 73 *SAUR* genes (*PmSAUR1~73*) through bioinformatics methods, systematically analyzing the physicochemical properties of the encoded proteins, chromosomal localization, phylogenetic relationships, gene structures, and cis-acting elements. Combined with transcriptome sequencing and molecular experiments, the drought stress response patterns of these genes were further elucidated. The results indicated that *PmSAUR* genes predominantly encode alkaline proteins, primarily localized in mitochondria and nuclei, with an uneven distribution across nine chromosomes, where tandem duplication serves as the primary mechanism driving family expansion. Phylogenetic analysis classified these genes into seven subfamilies, which include both conserved clades homologous to angiosperms and branches specific to *P. massoniana*. All members contain the Auxin_inducible conserved domain, with motif1 identified as the core essential motif. Promoter regions were enriched with MeJA (methyl jasmonate)-responsive (56%), ABA-responsive, and drought stress-related cis-elements. Under drought stress, 38 *PmSAUR* genes exhibited diverse temporal expression patterns. Four key genes (*PmSAUR14, PmSAUR28, PmSAUR54*, and *PmSAUR73*), which are localized in the nucleus and exhibit high expression specifically in male cones or roots, were identified. These genes exhibit an expression pattern consistent with an auxin-negative response (i.e., repressed by IAA and induced by drought) and display a distinctive response pattern characterized by drought-induced upregulation coupled with IAA-mediated downregulation. This mechanism may contribute to the drought adaptation strategies of *P. massoniana*, involving regulatory processes for aboveground reproduction and adaptation of the underground root system. This study represents the first effort to elucidate the evolutionary characteristics and drought response patterns of the *SAUR* gene family in *P. massoniana*, thereby addressing the existing research gap regarding the functions of *SAUR* genes in coniferous trees. Furthermore, it offers candidate gene resources and theoretical support for the molecular breeding of stress resistance in *P. massoniana*. In addition, two auxin-induced *SAUR* genes (*PmSAUR22* and *PmSAUR37*) were identified as contrasting examples, but the main focus of this study is on the four auxin-repressed genes.

## Simple Summary

*Pinus massoniana* Lamb is a key native tree species in southern China with great economic and ecological value, and seasonal drought severely restricts its growth and yield. The *SAUR* gene family is vital for plant growth and stress resistance, but relevant studies on coniferous trees are still insufficient. In this study, 73 *SAUR* genes were identified in *P. massoniana* through bioinformatics and molecular experiments to explore their functions in *P. massoniana* drought adaptation. We analyzed the gene structure, evolutionary characteristics and stress response elements of these genes and screened four core genes that can respond to drought stress. These unique genes may be involved in regulating the root growth and reproductive development of *P. massoniana* to help the tree adapt to drought environments. This study provides insights into the drought response patterns of the *SAUR* gene family in *P. massoniana*, fills the research gap for conifers, and provides valuable genetic resources and a theoretical basis for drought-resistant molecular breeding of *P. massoniana*.

## 1. Introduction

As a core phytohormone, auxin (IAA) plays a crucial role throughout the entire life cycle of plants, including embryonic development and organ formation, by regulating cell division, differentiation, and elongation. It also interacts with other hormones such as ABA and JA to integrate environmental signals, thereby enhancing abiotic stress tolerance in plants [1,2,3,4]. The *SAUR* gene family is one of the three major early auxin-responsive gene families (*Aux/IAA*, *GH3*, and *SAUR*) [5]. It responds rapidly to exogenous IAA induction within 2–5 min by inhibiting PP2C.D phosphatase activity, which helps maintain the phosphorylation status of plasma membrane H^+^-ATPase, thereby facilitating cell wall acidification, relaxation, and cell elongation. This regulatory pathway is high evolutionary conservation across the plant kingdom [6,7].

Since the first *SAUR* gene was cloned and identified from auxin-treated soybean (*Glycine max*) hypocotyls in 1987 [8], advances in genomic sequencing technology have enabled systematic genome-wide identification and characterization of the *SAUR* gene family across major evolutionary lineages, including mosses, gymnosperms, and angiosperms. The total number of *SAUR* members varies substantially among species: 66 in Chinese pine (*Pinus tabuliformis*) [9], 52 in pineapple (*Ananas comosus* L.) [10], 40 in oil palm (*Elaeis guineensis*) [11], 42 in ginkgo (*Ginkgo biloba*) [12], 58 in rice (*Oryza sativa*) [13], 64 in grape (*Vitis vinifera*) [14], 66 in watermelon (*Citrullus lanatus*) [15], 79 in Arabidopsis thaliana [16], 105 in black cottonwood (*Populus trichocarpa*) [17], and as many as 162 in peanut (*Arachis hypogaea*) [18]. Such marked variation in the number of *SAUR* genes reflects lineage-specific expansion of the *SAUR* family and is closely associated with plant life form, growth strategy, and ecological adaptation. Functional studies have demonstrated that the *SAUR* family acts as a multifunctional regulatory module governing plant growth, development, and stress adaptation. In developmental processes, *Arabidopsis AtSAUR63* promotes hypocotyl and stamen filament elongation by activating plasma membrane H^+^-ATPase [19]; cucumber *CsSAUR31* positively regulates root expansion and hypocotyl growth [20]; five *SAUR* genes in melon (*Cucumis melo*) are specifically and highly expressed in floral organs and ovaries, participating in stamen and pistil development as well as the temporal regulation of fruit ripening [21]. In stress responses, overexpression of wheat *TaSAUR75* enhances drought and salt tolerance in transgenic *Arabidopsis* by strengthening antioxidant capacity [22]; peanut *AhSAUR31* modulates drought adaptation by negatively regulating reactive oxygen species metabolism [18]; pineapple *AcoSAUR12/24/50* are significantly upregulated under combined salt and drought stress, representing promising stress-tolerance candidate genes [10]; coordinated expression of poplar *SAUR*34/54/67/91/97 improves seedling cold tolerance [17]; and Chinese pine *PtSAUR59/66* show remarkable upregulation under drought conditions [9]. Collectively, these findings indicate that *SAUR* genes function as core regulatory hubs that integrate hormone signaling and environmental cues to balance plant growth and stress tolerance.

However, current research on the *SAUR* gene family remains primarily focused on model angiosperms such as Arabidopsis and rice, with only limited exploration in gymnosperms, including a few species like *P. massoniana*, *Ginkgo biloba*, and *Cunninghamia lanceolata* [9,23,24,25]. The evolutionary characteristics, species-specific traits, and molecular mechanisms of stress response in conifer *SAUR* genes still require systematic elucidation.

*P. massoniana* is an evergreen coniferous tree belonging to the genus Pinus in the family Pinaceae. As a dominant native species in southern China, its plantation area accounts for over one-fifth of the total coniferous forest area nationwide [26,27]. It holds irreplaceable ecological and economic value in shelterbelt construction, rocky desertification control, and high-quality timber production [28,29]. Affected by global warming, seasonal droughts in southern China have become more frequent and prolonged, hindering the growth of *P. massoniana* plantations, reducing productivity, and severely limiting their potential value [30]. In recent years, the release of the chromosome-level genome of *P. massoniana* has provided data support for genome-wide analysis of the characteristics and functions of the *SAUR* gene family [26]. Based on this, this study focuses on *P. massoniana* as the research subject, systematically identifying the members of the *SAUR* gene family and analyzing their molecular characteristics. Combined with drought stress treatment, the expression patterns of this gene family are analyzed to screen for key drought-responsive genes and reveal their hormone regulation features. The aim is to elucidate the evolutionary patterns of the *SAUR* gene family in *P. massoniana* and its response mechanisms to drought stress, thereby filling the gap in functional studies of *SAUR* genes in conifers and providing candidate genes and theoretical foundations for the molecular breeding of stress-resistant *P. massoniana*.

## 2. Materials and Methods

### 2.1. Plant Materials

The test materials were two-year-old potted seedlings of *P. massoniana* cultivated at the State Key Laboratory of Tree Genetics and Breeding, Nanjing Forestry University. Seedlings with relatively uniform growth were selected for different exogenous hormone and drought treatments. The exogenous spray treatments were set as: 10 mM MeJA (Beijing Solarbio Science & Technology Co., Ltd., Beijing, China) and 10 mM indole-3-acetic acid (IAA, Beijing Solarbio Science & Technology Co., Ltd., Beijing, China). Needles were collected at 0 h, 3 h, 6 h, 12 h, and 24 h after treatment. For drought stress treatment, all seedlings were fully watered on day 0, and then irrigation was suspended to induce natural soil dehydration. The entire trial lasted 23 days, and needle samples were collected at six time points: 0 d, 3 d, 7 d, 12 d, and 20 d after the onset of drought stress, as well as 3 d after rewatering. The corresponding soil field water capacities at each sampling time point were recorded as follows: 0 d (83%), 3 d (79%), 7 d (64%), 12 d (48%), 20 d (31%), and 3 d after rewatering (76%). All samples were set up with three biological replicates. For the drought time-course experiment, three independent biological replicates (separate plantings) were used for each time point to ensure statistical independence. The collected needles were immediately frozen in liquid nitrogen and stored at −80 °C for further use.

### 2.2. Genome-Wide Identification of P. massoniana SAUR Genes

Reference gene sequences of *P. massoniana* were downloaded from GigaDB (https://gigadb.org/dataset/102688, accessed on 16 October 2025) to identify members of the *SAUR* gene family. The *SAUR* protein sequences of *A. thaliana* were obtained from the UniProt database (https://www.uniprot.org/, accessed on 18 October 2025) and used as seed sequences to search for *SAUR* gene family members in the *P. massoniana* reference sequences. Simultaneously, the hidden Markov model (PF02519) of the *SAUR* protein domain was downloaded from the Pfam database (http://pfam.xfam.org/, accessed on 18 October 2025). A BLAST search was performed on the whole genome sequence of *P. massoniana* using Tbtools-II version 2.472 software to obtain the protein sequences of candidate genes. Conserved domain predictions were performed using the CDD database from NCBI (https://www.ncbi.nlm.nih.gov/Structure/cdd/wrpsb.cgi, accessed on 25 October 2025). Sequences lacking the *SAUR* protein domain were removed, and the remaining *SAUR* proteins were aligned using MEGA11 software, with duplicate sequences subsequently eliminated.

### 2.3. Basic Physicochemical Properties, Amino Acid Sequence Alignment, and Synteny Analysis of the SAUR Gene in P. massoniana

The amino acid sequence length, molecular weight, and isoelectric point of the identified *SAUR* gene family members were predicted using the ExPASy proteomics server (http://ca.expasy.org/, accessed on 29 October 2025). Subcellular localization predictions were conducted through the Cell-PLoc website (http://www.csbio.sjtu.edu.cn/bioinf/Cell-PLoc/, accessed on 11 November 2025). The chromosomal localization of *PmSAUR* gene family members was analyzed using Tbtools-II. Additionally, the whole-genome collinearity map of Pinus massoniana and Pinus tabuliformis was constructed with the MCScanX tool integrated into TBtools-II

### 2.4. Chromosomal Localization Prediction and Naming of Members of SAUR Family in P. massoniana

The GFF file for genome annotation of *P. massoniana* is available from the GigaDB database (https://gigadb.org/dataset/102688, accessed on 15 December 2025). The gene positions visualized from the GTF/GFF module, along with the gene IDs of the *SAUR* family members, were incorporated into the GFF file. This information was subsequently utilized to create chromosomal location maps using Tbtools-II.

### 2.5. Conserved Motifs and Domain Analysis of SAUR Proteins in P. massoniana

Utilize the MEME Suite (http://meme-suite.org/tools/meme, accessed on 3 December 2025) to conduct a conservative sequence analysis of PmSAUR proteins. Additionally, employ the Tbtools software to create a comprehensive diagram that includes conserved motifs, domains, and the phylogenetic tree of PmSAUR proteins. The upstream 2000 bp promoter sequences of *PmSAUR* genes were extracted with Tbtools-II and uploaded to the PlantCARE program (http://bioinformatics.psb.ugent.be/webtools/plantcare/html/, accessed on 3 December 2025) for the prediction and analysis of promoter cis-acting elements. The results were visualized using Tbtools-II. A phylogenetic tree comprising *PmSAUR*s and *AtSAURs* was constructed using MEGA 11.0 software, employing the neighbor-joining method with bootstrap values set to 1000, and subsequently edited and enhanced using the online tree visualization tool iTOL (https://itol.embl.de/, accessed on 15 December 2025).

### 2.6. Expression Profile Analysis of SAUR Gene Family in P. massoniana

Download the drought transcriptome data of *P. massoniana* (PRJNA693351) subjected to mild and severe drought followed by rewatering treatment from the NCBI SRA (National Center for Biotechnology Information Short Read Archive) database (https://www.ncbi.nlm.nih.gov.sra, accessed on 24 December 2025). The gene expression data of *SAUR* in *P. massoniana* were extracted using Galaxy (https://usegalaxy.org/, accessed on 24 December 2025), TPM values were calculated, and expression profiles were generated using Tbtools-II software. Transcriptome sequencing was conducted using needle tissues of *P. massoniana* collected on days 0, 3, 6, 9 and 12 of drought treatment. Gene expression abundance was calculated as TPM. For heatmap plotting, expression values were row-normalized to reduce inherent expression differences among genes and facilitate the comparison of temporal expression trends. In this study, 38 *PmSAUR* genes with apparent expression changes during drought stress were selected for expression pattern analysis.

### 2.7. Subcellular Localization Analysis

The open reading frames (ORFs) of four *PmSAUR* genes, which lack stop codons, were cloned into the pCAMBIA-1302-GFP vector using primers listed in Table S3 Following PCR confirmation, positive Agrobacterium clones were cultured in LB medium supplemented with kanamycin (50 mg/L) and rifampicin (25 mg/L) until the optical density (OD600) reached 0.6. The bacterial suspension was then combined with the P19 strain (an RNA silencing suppressor) in an infiltration buffer containing 200 µM acetosyringone (AS), 10 mM MgCl2, and 10 mM 2-(N-morpholino)ethanesulfonic acid (MES). This mixture was subsequently infiltrated into 4-week-old tobacco leaves. After infiltration, the plants were kept in darkness for 48 h to promote protein expression. Finally, GFP fluorescence was observed using an LSM710 confocal laser scanning microscope (Zeiss, Jena, Germany).

### 2.8. RNA Extraction and RT-qPCR Analysis

Total RNA was isolated from *P. massoniana* using the FastPure Plant Total RNA Isolation Kit (RC401, Vazyme Biotech, Nanjing, China) following the manufacturer’s instructions. RNA concentration and purity were determined using a NanoDrop 2000 spectrophotometer (Thermo Fisher Scientific, Waltham, MA, USA), while RNA integrity was assessed through 1% agarose gel electrophoresis. First-strand cDNA synthesis was conducted using the All-in-One gDNA Removal and cDNA Synthesis Kit (AT311, Trans-Gen Biotech, Beijing, China). Gene-specific primers for real-time quantitative RT-PCR were designed using Primer 5.0 software (Table S2). Amplification was performed in a 10 µL reaction volume with SYBR Green Master Mix (11184ES03, Yeasen Biotech, Shanghai, China), which included 1 µL of 20-fold diluted cDNA, 5 µL of Master Mix, 0.4 µL of each primer (10 µM), and 3.2 µL of ddH_2_O. The thermal cycling conditions consisted of an initial denaturation at 95 °C for 2 min, followed by 40 cycles of 95 °C for 10 s and 60 °C for 30 s; the remaining steps were conducted according to the instrument’s default protocol. Amplification specificity was confirmed by analyzing the melting curve. The α-tubulin (TUA) gene served as the internal reference. Each sample included three biological replicates, with three technical replicates per biological replicate. Relative gene expression levels were calculated using the 2^−∆∆Ct^ method [∆CT = CT target − CT TUA; ∆Ct = ∆Ct Target − ∆Ct CK]. Statistical significance was evaluated using one-way ANOVA followed by Tukey’s honestly significant difference (HSD) post hoc test (*p* < 0.05). Effect sizes (Cohen’s d) and 95% confidence intervals are provided in Table S4.

## 3. Results

### 3.1. Identification of SAUR Genes in P. massoniana

Through conserved domain prediction, multiple sequence alignment, and the removal of redundant sequences, a total of 73 *SAUR* genes were identified. These genes were designated *PmSAUR1–73* according to their sequential order on the chromosomes (from chromosome 2 to chromosome 12) (Table S1). Among the 73 PmSAUR proteins, the number of amino acids ranged from 107 to 258, with predicted molecular weights varying between 12.38 and 29.38 kDa. The isoelectric point (pI) values ranged from 5.48 to 10.34. The detailed ranges of protein molecular weights and isoelectric point values are listed in Table S1. Notably, 66 *SAUR* genes encode proteins with pI values greater than 7, indicating that 76% of *SAUR* genes encode alkaline proteins that function in alkaline subcellular microenvironments. According to subcellular localization predictions by CELLO and WolfPSORT, 61.64% of *SAUR* proteins are predicted to be localized in mitochondria, followed by 30.14% in the nucleus, with minor distributions also observed in the cytoplasm, plasma membrane, and extracellular space (Table S1).

### 3.2. Chromosomal Distribution of SAUR Genes

**Figure 1:**
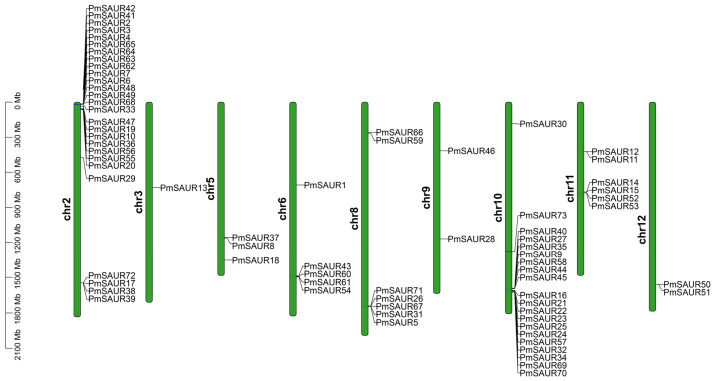
Chromosomal distribution of *PmSAUR* gene.

The locations of the *PmSAUR* genes were obtained from the genome annotation file. A total of 73 *PmSAUR* genes were unevenly distributed across 9 chromosomes. Notably, no *SAUR* genes were identified on chromosomes 1, 4, or 7. Chromosome 2 harbored the highest number of genes (27), with 22 of these genes forming a clustered distribution on its upper arm. Chromosome 10 contained 20 genes, including 19 that were clustered on its lower arm. Chromosome 11 had 7 genes, chromosome 5 had 3 genes, chromosome 6 carried 5 genes, chromosome 8 possessed 7 genes, and chromosome 12 had 6 genes. Both chromosomes 9 and 12 each contained 2 genes, while chromosome 3 had the fewest, with only 1 gene (Figure 1).

**Figure 2:**
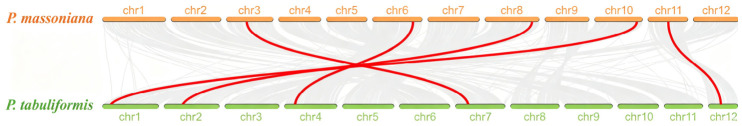
Collinearity analysis of *SAUR* genes between *P. massoniana* and *P. tabuliformis*. The red line represents collinear relationship of *SAUR*.

To investigate the evolutionary conservation and syntenic relationships of *SAUR* genes between *P. massoniana* and its congener *P. tabuliformis*, a cross-species collinearity analysis was performed. As shown in Figure 2, the chromosomes of the two species exhibit extensive genome-wide collinearity, as indicated by the gray background lines, reflecting the conserved genomic structure within the Pinus genus [9,23].

The twelve chromosomes of *P. massoniana* and *P. tabuliformis* showed relatively high collinearity, indicating conserved genomic structure and a close genetic relationship between the two species [26,27]. No large-scale chromosomal rearrangement events were inferred based on the current collinearity comparison. Several interchromosomal syntenic blocks were observed: large collinear segments observed between *P. massoniana* chromosomes 3, 6, 8, and 10 and *P. tabuliformis* chromosomes 1, 2, 4, and 7, respectively. In addition, potential reciprocal translocation events were found between chromosomes 11 and 12 (Figure 2) [31].

Notably, several pairs of *SAUR* genes were identified as syntenic orthologs, which are highlighted by the red lines connecting their corresponding chromosomal positions. These collinear *SAUR* gene pairs are distributed across multiple chromosomes in both species, suggesting that the chromosomal locations of these genes have been largely conserved following the divergence of *P. massoniana* and *P. tabuliformis* [12,13]. The presence of these syntenic orthologs indicates that the *SAUR* gene family evolved prior to the speciation of these two pine species, and that these genes have likely retained their core functions related to auxin signaling and environmental responses during long-term evolution [16,32].

### 3.3. Phylogenetic Analysis, Conserved Motifs and Domains of PmSAUR Proteins

**Figure 3:**
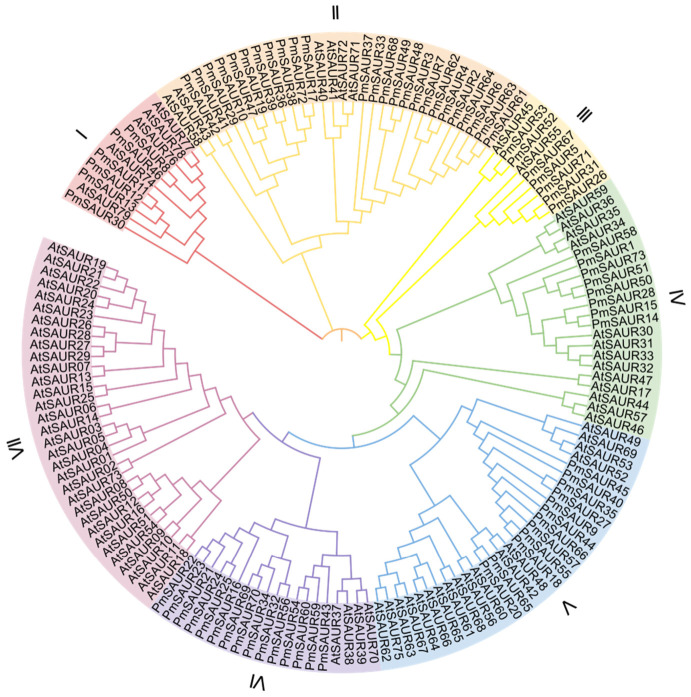
Phylogenetic analysis of SAUR protein sequences in *P. massoniana* and *A. thaliana*.

**Figure 4:**
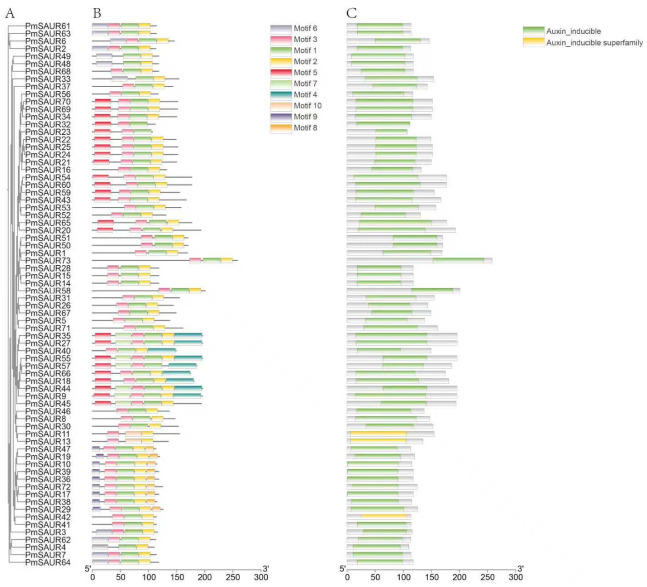
Analysis of gene structure characteristics of *PmSAUR* genes. (**A**) Neighbor-joining (NJ) tree containing 73 *SAUR* genes from *P. massoniana*. (**B**) Architecture of conserved motifs: Different colored boxes represent different conserved motifs. (**C**) Gene structures of *PmSAUR* genes: green represents the auxin-inducible domain, and yellow lines represent the auxin-inducible superfamily.

Through phylogenetic analysis involving both Arabidopsis thaliana and *P. massoniana*, all PmSAURs were classified into seven subfamilies. On branch I, PtSAUR31, PtSAUR56, PtSAUR57, and PtSAUR60 were the first to diverge from the phylogenetic tree, likely indicating a specificity to *P. massoniana*. In branch II, eleven PmSAUR proteins clustered with five Arabidopsis proteins, namely AtSAUR2, AtSAUR16, AtSAUR28, AtSAUR40, and AtSAUR74, with PtSAUR52 and AtSAUR28 forming a closely related subclade. Branch III comprised twenty PmSAUR proteins and fourteen AtSAUR proteins, where AtSAUR77 and PtSAUR66 exhibited a closer evolutionary proximity. PtSAUR22, PtSAUR28, PtSAUR29, PtSAUR30, PtSAUR37, PtSAUR38, and PtSAUR61 clustered with AtSAUR26, AtSAUR29, AtSAUR42, AtSAUR52, and AtSAUR73 to form branch IV. Branches V and I exclusively contained PmSAUR proteins, suggesting that they possess highly conserved gene structures within *P. massoniana*. On branch VII, PtSAUR3, PtSAUR10, PtSAUR29, PtSAUR40, and PtSAUR53 clustered with thirty-two Arabidopsis proteins. Branches II, III, IV, and VII all contain SAURs from both Arabidopsis and *P. massoniana*, indicating that the SAUR proteins in these branches of *P. massoniana* have progressively evolved towards angiosperms. Therefore, *P. massoniana* possesses both SAUR proteins that are closely related to those in angiosperms and unique SAUR proteins that diverged earlier (Figure 3). According to the MEME program analysis (Figure 4), a total of ten motifs were identified in the *SAUR* family of *P. massoniana*. Most SAUR proteins from *P. massoniana* contain motifs 1, 2, and 3. With the exception of PmSAUR11 and PmSAUR13, all SAUR proteins possess motif 1, suggesting that this motif may be essential for SAUR proteins in *P. massoniana*. SAUR proteins containing motifs 5, 6, or 9 cluster together, indicating that these motifs may have distinct functions. Most genes with motif 5 also possess motif 4, implying that these two motifs may work together to perform a specific function. All *SAUR* family proteins in *P. massoniana* contain the Auxin_inducible conserved domain (which belongs to the Auxin_inducible superfamily).

### 3.4. Analysis of Cis-Acting Elements in the PmSAUR Promoter

To further explore the potential roles of *PmSAUR*s in the plant regulatory network, we analyzed the cis-acting elements in the promoter regions of 73 *PmSAUR* genes using the PlantCARE online tool. These elements were broadly classified into three groups, encompassing abiotic stress responses, phytohormone responses, and plant growth and development regulation. Accumulating evidence has indicated that numerous cis-elements are closely implicated in abiotic stress adaptation, phytohormone signaling, and plant growth and developmental processes (Figure S1).

**Figure 5:**
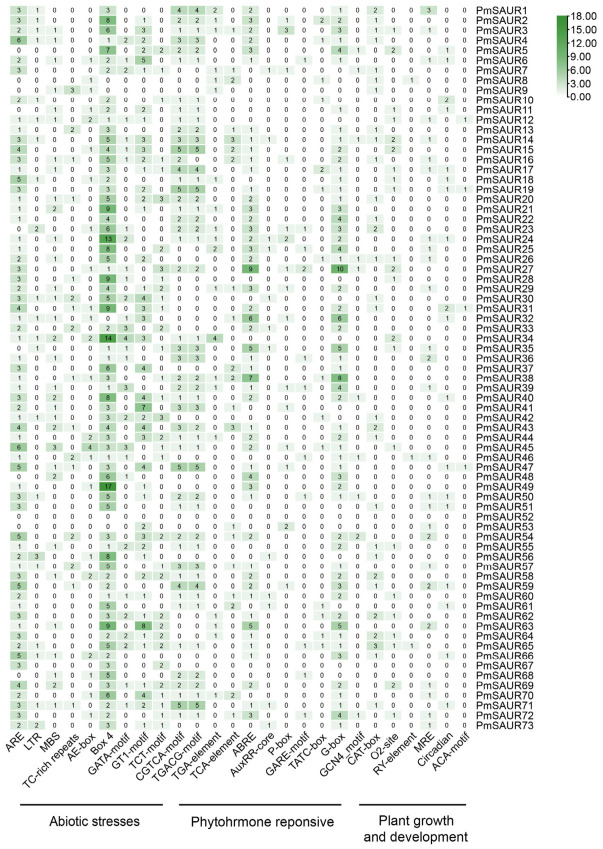
Predictive analysis of *SAUR* gene family promoter cis-element in *P. massoniana*. The numbers in the heat map indicate the counts of each cis-acting element in the promoter region of each *PmSAUR* gene. Green indicates higher counts and white indicates lower counts (the color gradient from white to green represents low to high counts).

A total of nine cis-regulatory elements were identified within the abiotic stress response category, the majority of which were well-characterized stress-related motifs such as ARE, Box4 and GT1-motif. For cis-elements implicated in plant growth and development, seven distinct elements were detected, with the predominant ones being ATCT-motif, G-box, GATA-motif and GT1-motif, all of which are known to mediate light-responsive signaling pathways in plants. Additionally, ten cis-regulatory elements were classified as phytohormone-responsive elements, including ABRE designated as an abscisic acid-responsive element, TGACG-motif as a methyl jasmonate-responsive element, TCA as a salicylic acid-responsive element, P-box as a gibberellin-responsive element and TGA-element as an auxin-responsive element. Notably, methyl jasmonate (MeJA)-responsive cis-regulatory elements accounted for the highest proportion among all phytohormone-responsive elements in *PmSAUR*s, with 202 such cis-acting elements distributed across all analyzed promoters. Further cis-element analysis corroborated that these regulatory elements perform crucial functions in modulating plant growth and developmental processes as well as adaptive responses to abiotic stresses (Figure 5).

### 3.5. Expression Patterns of SAUR Gene Family in P. massoniana Under Drought Stress

**Figure 6:**
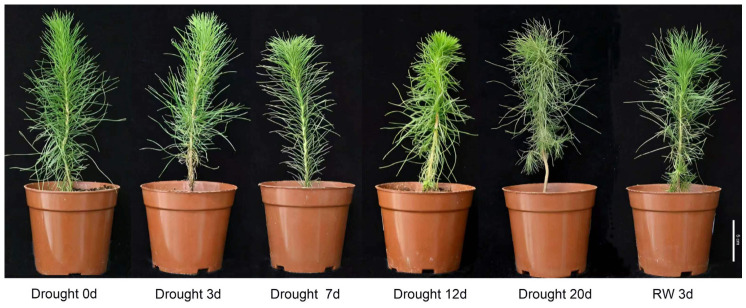
Phenotypic changes of *P. massoniana* seedlings under drought stress.

To visually demonstrate the effects of drought stress on the growth of *P. massoniana*, this study observed and recorded the phenotypic changes of *P. massoniana* seedlings under drought stress for 0, 3, 7, 12, and 20 days as well as after 3 days of rewatering, and completed corresponding image collection (Figure 6). The results showed that compared with the normally growing control group, the overall growth vigor of plants decreased significantly with the prolongation of drought stress, and needles gradually exhibited typical drought injury characteristics such as wilting, curling and chlorosis. The above phenotypic changes confirmed that the drought gradient established in this study can effectively simulate natural drought conditions, laying a reliable phenotypic foundation for in-depth analysis of the drought-responsive expression patterns and functional verification of the *SAUR* gene family in *P. massoniana*. While these phenotypic observations provide a reliable basis for further analysis, future studies will incorporate quantitative physiological indicators, such as chlorophyll content, malondialdehyde (MDA) levels, and relative water content, to more comprehensively evaluate the drought tolerance of *P. massoniana* seedlings.

**Figure 7:**
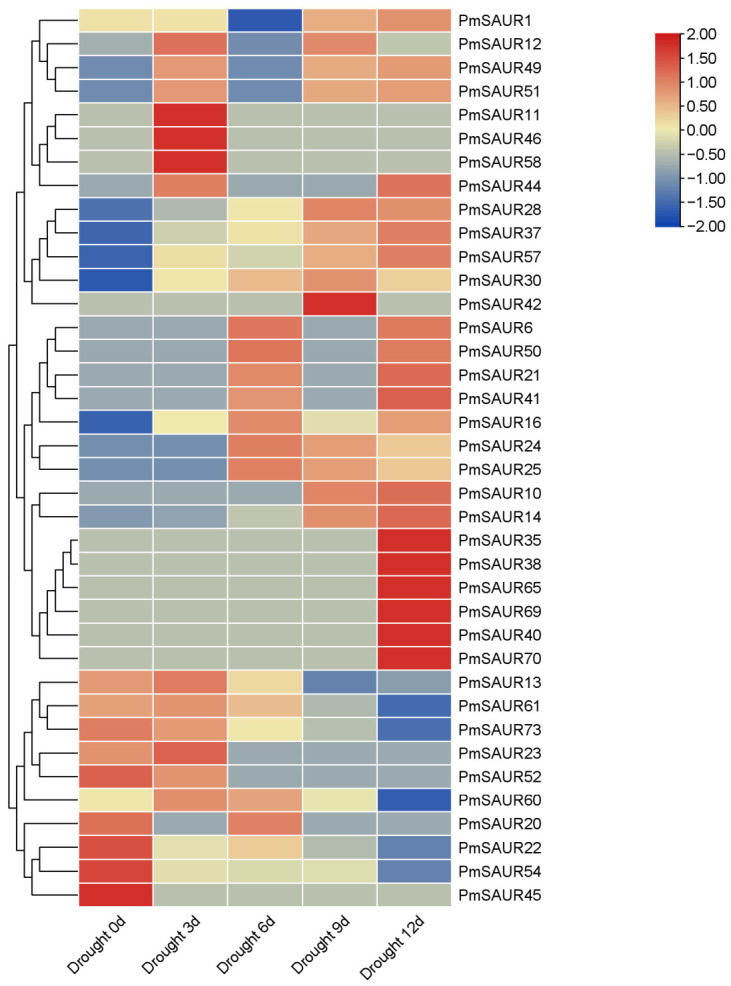
Temporal expression patterns of 38 *PmSAUR* genes in response to drought stress in *P. massoniana.* Gene expression levels were calculated based on transcriptome data of needle tissues collected at different drought treatment stages. The heatmap shows row-normalized expression values of the selected genes. The 38 *PmSAUR* genes displayed obvious expression variations under drought conditions. Red indicates higher expression and blue indicates lower expression. (The color gradient from blue to red represents low to high relative expression levels, respectively.).

Based on transcriptome data, we analyzed the expression levels of 38 *PmSAUR* genes under drought stress using a heatmap (Figure 7). Most *PmSAUR* genes exhibited significant differential expression during drought stress, suggesting that this gene family is potentially involved in the drought stress response mechanism of *P. massoniana*. Expression trend analysis revealed that *PmSAUR* family members displayed diverse expression patterns under drought stress. Specifically, there were early rapid-response genes: a total of 11 *PmSAUR* genes showed a significant increase in expression at the early stage of drought stress (3 d), with expression peaks observed at 3–6 d, and their expression levels gradually decreased as drought stress persisted (9–12 d). There were also mid-term sustained regulatory genes: a total of 9 *PmSAUR* genes were continuously and significantly up-regulated with the prolongation of drought stress (0–12 d), without an obvious down-regulation trend throughout the entire stress period. In addition, there was the late-repair up-regulated type: a total of 8 *PmSAUR* genes showed no significant changes in expression at the early stage of drought stress (0–6 d), but were significantly induced and up-regulated at the late stage of stress (9–12 d).

### 3.6. Expression Patterns Under Drought Stress and Plant Hormone Action

**Figure 8:**
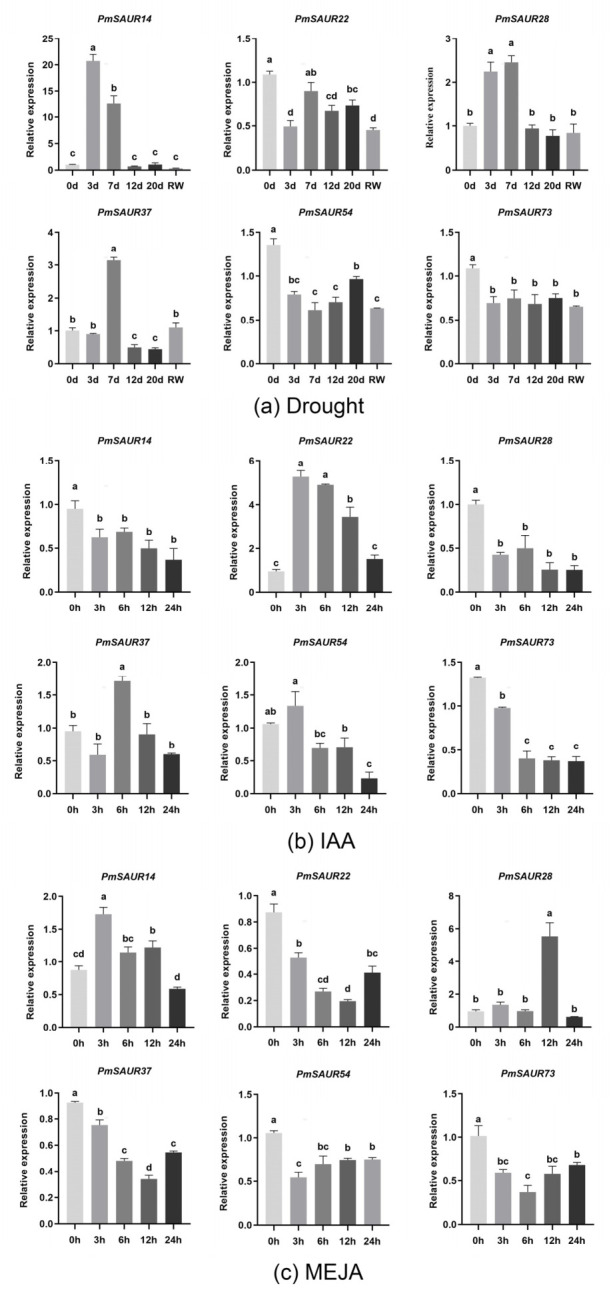
Expression levels of six *PmSAUR*s under different treatments. (**a**) Expression patterns of six *SAUR* genes in *P. massoniana* under drought stress. 0 d, 3 d, 7 d, 12 d, and 20 d represent the time points after drought treatment, respectively. RW indicates rewatering after 3 days. (**b**) Expression patterns of six *SAUR* genes in *P. massoniana* under auxin treatment. 0 h, 3 h, 6 h, 12 h, and 24 h represent the time points after IAA treatment, respectively. (**c**) Expression patterns of six *SAUR* genes in *P. massoniana* under MeJA treatment. 0 h, 3 h, 6 h, 12 h, and 24 h represent the time points after MeJA treatment, respectively. Analysis of *PmSAUR*s gene expression under drought treatment based on RT-qPCR.The relative expression levels of four *PmSAUR* genes were detected by RT-qPCR.Error bars represent the standard deviation of three biological replicates. Values with different letters within the same column indicate significant differences.

Based on the research background of promoter cis-acting elements and the *SAUR* gene family, combined with the core theme of drought stress adaptation in *P. massoniana*, seedlings were subjected to exogenous IAA and MeJA treatments, aiming to analyze the hormone response characteristics and drought-related regulatory functions of four key *PmSAUR* genes (*PmSAUR14/28/54/73*), with corresponding results shown in Figure 8b.

Under IAA treatment, all four target genes displayed significant expression suppression, which was sharply different from the typical IAA-induced upregulation pattern of most classical *SAUR* genes. This result suggests that these four genes may represent a unique subclass of auxin-negative-responsive members within the *PmSAUR* gene family.

Notably, this reverse regulatory pattern characterized by drought induction and IAA inhibition is highly adaptive to drought stress: According to a previous study on *P. massoniana*, endogenous IAA levels in pine seedlings usually decrease significantly [33,34], which can relieve the suppression of these four *PmSAUR* genes and trigger their expression upregulation, thereby mediating the drought resistance response of *P. massoniana*.

**Figure 9:**
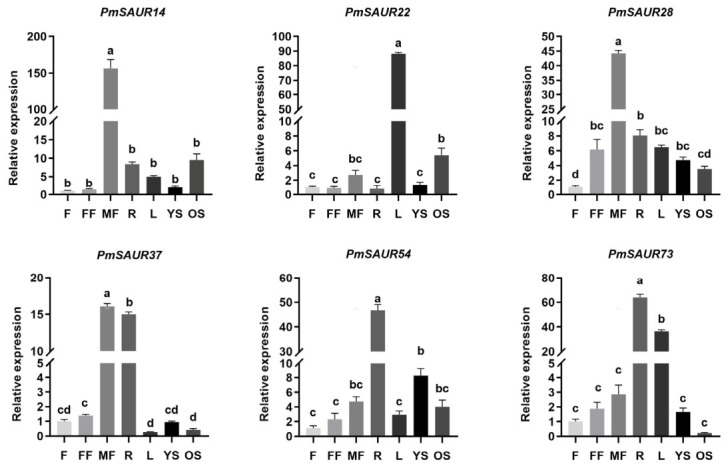
Relative expression levels of four *PmSAUR* in different tissues. Expression analysis of *PmSAUR*s in different tissues based on RT-qPCR. The relative expression levels of four *PmSAUR* genes were detected by RT-qPCR. Tissues: female cones (FF), male cones (MF), cones (F), young stems (YS), old stems (OS), roots (R), and needles (L). Error bars indicate the standard deviation of three biological replicates. Values with different letters within the same column indicate significant differences.

Specifically, *PmSAUR54* and *PmSAUR73* show expression patterns associated with root system remodeling and drought adaptation regulation, as supported by their root-preferential expression (Figure 9) and significant upregulation under drought stress (Figure 8a) [35,36], while *PmSAUR14* and *PmSAUR28* participate in the regulation of male cone development. This functional differentiation and reverse expression pattern form the core molecular mechanism underlying the growth-defense trade-off strategy of *P. massoniana* in response to drought stress, fully reflecting the unique hormone response characteristics and functional diversification of the *SAUR* gene family in woody plants (Figure 8A).

To complement the regulatory mechanism of the *PmSAUR* family, we further screened two drought-responsive *PmSAUR* members (*PmSAUR22* and *PmSAUR37*) that are positively induced by auxin. In addition to the four core auxin-negative-responsive genes, we also identified two auxin-induced *SAUR* genes (*PmSAUR22* and *PmSAUR37*) as contrasting examples for comparison. qPCR results showed that both genes were markedly upregulated under exogenous IAA treatment: *PmSAUR22* reached the expression peak at 3–6 h with more than 5-fold increase, and *PmSAUR37* peaked at 6 h with approximately 1.7-fold higher expression than the control, confirming that they belong to the typical auxin-induced *SAUR* subfamily. These two auxin-induced genes may coordinate with the four auxin-negative-responsive genes to jointly regulate the balance between plant growth and drought adaptation, enriching the regulatory network of *PmSAUR* genes in hormone signal crosstalk.

Under MeJA treatment, the above six key *PmSAUR* genes exhibited distinct differential expression responses, which were closely related to drought stress and MeJA signal transduction: *PmSAUR*14 was significantly induced at 3 h after treatment, and *PmSAUR28* showed obvious upregulation at 12 h, whereas *PmSAUR54* and *PmSAUR73* presented remarkably decreased expression levels. For the two newly identified auxin-induced members, *PmSAUR22* was rapidly and persistently inhibited by MeJA, while *PmSAUR37* was slightly induced at 6 h and then gradually declined. Under normal growth conditions, MeJA treatment was applied to simulate the activation of jasmonate signaling that occurs during drought stress. This differential temporal expression pattern reveals the high temporal and functional specificity of these key *PmSAUR* genes in response to MeJA signaling under conditions relevant to drought stress, and this expression characteristic is highly consistent with the distribution features of MeJA-responsive cis-acting elements in their promoter regions (Figure 8c).

### 3.7. Expression Patterns in Different Tissues

The RT-qPCR analysis presented in Figure 9 illustrates the expression patterns of four *PmSAUR* genes across seven different tissues: FF (female cone), MF (male cone), F (cone), YS (young stem), OS (old stem), L (needle), and R (root).

It is evident that *PmSAUR14* and *PmSAUR28* are predominantly expressed in male cones, whereas *PmSAUR54* and *PmSAUR73* are primarily expressed in roots. By contrast, *PmSAUR22* shows the highest expression in needles, with significantly lower levels in other tissues, while *PmSAUR37* exhibits preferential expression in both male cones and roots. These expression patterns align closely with the division of labor strategy between aboveground reproductive regulation and underground root adaptation, and photosynthetic tissue response under drought stress.

Collectively, these tissue-specific patterns reveal a coordinated functional division among the six *PmSAUR* genes: reproductive-preferential (*PmSAUR14/28*), root-preferential (*PmSAUR54/73*), leaf-preferential (*PmSAUR22*), and dual-tissue-preferential (*PmSAUR37*) expression collectively contribute to the drought response strategy of *P. massoniana*, suggesting that these *SAUR* genes may play significant roles in the mechanisms of drought response.

### 3.8. Subcellular Localization Assay

**Figure 10:**
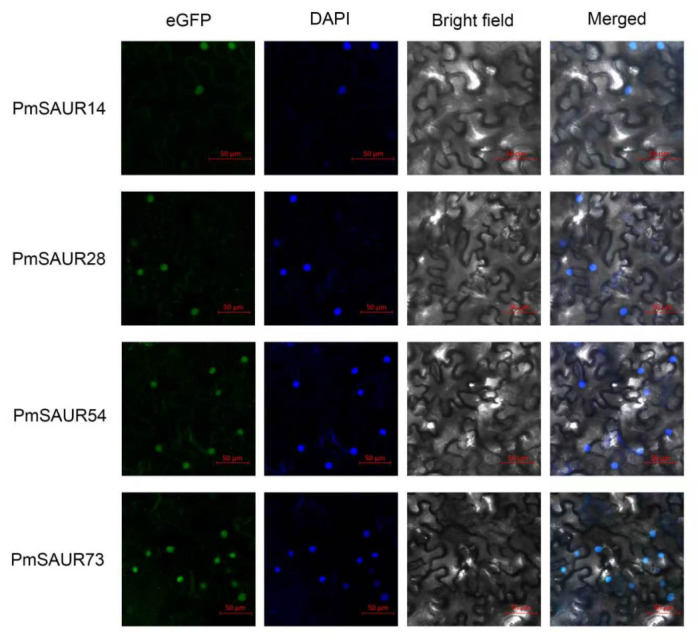
Subcellular localization analysis of PmSAUR protein. The recombinant vector 1305::*PmSAUR*-eGFP was transiently expressed in Nicotiana benthamiana leaves via agroinfiltration. The green fluorescence signal of eGFP was captured using a laser scanning confocal microscope (leftmost panel). As a fluorescent dye, DAPI specifically binds to AT-rich regions of double-stranded nuclear DNA and produces blue fluorescence in the nucleus (second panel from the left). Bright-field transmitted light images of the identical cells were acquired (second panel from the right), followed by the merged image of the three channels (rightmost panel). Scale bar = 50 μm. Quantitative co-localization analysis yielded a Pearson’s correlation coefficient of 0.89 ± 0.04 and a Mander’s overlap coefficient of 0.92, verifying strong co-localization between the GFP fluorescence signal and DAPI-stained cell nuclei.

Subcellular localization prediction showed that the four *PmSAUR* proteins screened based on expression heatmap data all exert regulatory functions in the nucleus. To verify the accuracy of the prediction, subcellular localization experiments were performed on these four *PmSAUR* genes. The results (Figure 10) revealed that the eGFP fluorescence signal was specifically distributed only in the nucleus, confirming that all four PmSAUR proteins are localized to the nucleus.

## 4. Discussion

As a core native coniferous species in southern China, *P. massoniana* faces increasing limitations in its growth and productivity due to seasonal drought. Exploring the molecular regulatory pathways underlying its drought stress response is essential for advancing stress-resistant breeding. The *SAUR* gene family, which serves as a central module in early auxin response, is presumed to participate in balancing plant growth and stress resistance. However, its evolutionary characteristics and potential functional mechanisms in conifers remain unclear. Based on the chromosome-level genome of *P. massoniana*, this study performed genome-wide identification and systematic analysis of the *SAUR* gene family. By integrating transcriptome sequencing and molecular experiments, it preliminarily characterized the putative drought response characteristics mediated by this family in *P. massoniana*. These findings not only may enrich the evolutionary and functional research of the *SAUR* gene family in gymnosperms but also provide valuable clues into the molecular regulatory network underlying drought adaptation in coniferous trees [31].

Subcellular localization prediction showed that 61.64% of PmSAUR proteins are predicted to be in mitochondria and 30.14% in the nucleus, indicating diverse possible localizations across the family. For the four key drought-responsive PmSAUR proteins (PmSAUR14/28/54/73), experimental validation confirmed that they are exclusively localized to the nucleus (Figure 10). Physicochemical property and subcellular localization analyses showed that 76% of PmSAUR proteins are alkaline, with a primary localization in mitochondria (61.64%) and the nucleus (30.14%). These characteristics closely align with those of the SAUR proteins from *P. tabuliformis* (76% alkaline, 74% nuclear localization) [37,38], suggesting the evolutionary conservation of both physicochemical properties and subcellular localization among conifer SAUR proteins. The nuclear localization of these four key proteins may imply their potential role as transcriptional regulators integrating auxin signals. The predicted mitochondrial localization for other members suggests that different PmSAUR proteins might function in distinct subcellular compartments, possibly reflecting functional diversification.

In terms of the overall molecular characteristics and evolutionary patterns of the gene family, this study identified 73 *PmSAUR* genes, a number that is intermediate between the gymnosperm *P. tabuliformis* (66) [8] and the angiosperm *A. thaliana* (79) [6], and significantly fewer than the polyploid angiosperm peanut (162) [13]. This quantitative feature likely reflects fundamental differences in genome evolutionary rates, ploidy levels, and expansion strategies between conifers and angiosperms. It also suggests that the *SAUR* gene family in gymnosperms tends to be conservatively retained during evolution rather than undergoing the rapid expansion observed in angiosperms. Chromosomal localization analysis showed that *PmSAUR* genes are unevenly clustered across nine chromosomes, with chromosomes 2 and 10 harboring 27 and 20 genes, respectively. The clustered regions exhibited high sequence homology among genes, supporting the possibility that tandem duplication may serve as the primary driving force for *PmSAUR* family expansion. This mechanism is highly consistent with the *SAUR* gene expansion patterns observed in plants such as *P. tabuliformis*, cucumber, and watermelon [8,12,19], demonstrating the evolutionary conservation of this gene family within the plant kingdom. Furthermore, the 12 chromosomes of *P. massoniana* and *P. tabuliformis* exhibit a high degree of 1:1 synteny overall, with a conserved genomic structure, suggesting their close genetic relationship. No large-scale chromosomal rearrangements were detected during their evolution, with only localized interchromosomal rearrangements observed. These findings may provide a genomic foundation for comparative genomic studies of the *SAUR* gene family in Pinus species, as well as valuable clues for deciphering the evolutionary patterns and common mechanisms of stress adaptation in pine species [31].

Phylogenetic analysis showed the dual evolutionary characteristics of the *PmSAUR* gene family, exhibiting both conservation and species specificity. Clades II, III, IV, and VII clustered with AtSAUR proteins to form conserved branches, suggesting that genes in these clades may potentially retain the core functions of plant *SAUR* genes in regulating cell elongation and organ development, consistent with the conserved branch features observed in angiosperms such as pineapple and peanut [10,13]. In contrast, clades A and E appear to be unique subfamilies specific to *P. massoniana*, exhibiting low amino acid sequence homology with other species. This is hypothesized to result from species-specific differentiation during long-term adaptation to arid and nutrient-poor habitats in southern regions, a pattern that aligns closely with the evolutionary trajectory of the *SAUR* family in *P. tabuliformis* [8]. These findings may reflect the evolutionary strategy of gymnosperm *SAUR* genes, which maintain fundamental functions while undergoing specific differentiation to adapt to unique environmental conditions. Conserved motif and domain analysis showed that all PmSAUR proteins, except for two members, contained motif 1, and the entire family possessed the Auxin_inducible conserved domain, which is entirely consistent with the conserved structural characteristics of *SAUR* genes in pineapple, cucumber, peanut, and other plants [10,12,13], thereby supporting the accuracy of gene identification in this study. Meanwhile, motif 5, motif 6, and motif 9 were found exclusively in specific subfamilies, with genes containing motif 5 often accompanied by motif 4. This suggests that the combinatorial regulation of different motifs may potentially serve as an important molecular basis for the functional differentiation of *PmSAUR* genes, providing potential candidate targets for subsequent research on the functional specificity of *SAUR* genes [39].

The analysis of promoter cis-acting elements offers important clues into the regulatory network of *PmSAUR* genes. The results showed an enrichment of MeJA-responsive elements (56%), along with ABA-responsive, auxin-responsive, and drought stress-related elements in the promoter regions. Notably, the proportion of MeJA-responsive elements was significantly higher than that of the other elements, which aligns with the predominant presence of MeJA-related elements in the promoters of PtSAUR genes [8]. This suggests a potential regulatory role of the jasmonic acid (JA) signaling pathway in the regulation of conifer *SAUR* genes, contrasting with the auxin-responsive element-rich promoters of pineapple *SAUR* genes [10] and the abiotic stress-responsive element-enriched promoters of peanut *SAUR* genes [13]. These findings likely imply adaptive characteristics of *PmSAUR* genes in hormone signal integration and stress response [40], particularly focusing on the possible coordination of JA/ABA signaling pathways with drought stress signals, thereby providing valuable hints into the regulatory networks underlying the putative regulatory pathways of *PmSAUR* genes in response to drought stress. It should be explicitly acknowledged that the presence of cis-acting elements in the promoter does not equate to the actual regulatory function of these elements. This is a limitation of the present study, as the real regulatory roles of the identified cis-acting elements remain to be verified by further molecular experiments.

The core breakthrough of this study lies in elucidating the unique drought stress response patterns and hormonal regulation mechanisms of the *SAUR* gene family in *P. massoniana*. Transcriptome analysis showed that 38 *PmSAUR* genes were involved in drought stress responses, exhibiting diverse temporal expression patterns, including rapid early response, sustained mid-term regulation, and late repair response. These findings are consistent with the drought response features observed in wheat TaSAUR75 and pineapple *AcoSAUR12/24/50* genes [10,11], supporting the possibility of a conserved function of the *SAUR* gene family in plant drought stress responses [41]. The four key genes further screened (*PmSAUR14/28/54/73*) were all localized in the nucleus and exhibited strict tissue-specific expression patterns. *PmSAUR14* and *PmSAUR28* were specifically highly expressed in male cones, whereas *PmSAUR54* and *PmSAUR73* were predominantly expressed in roots. This labor division strategy, regulating aboveground reproductive organs while adjusting belowground root systems, appears to be consistent with the potential survival strategy of *P. massoniana*, which balances reproductive success and water uptake efficiency in arid environments. This characteristic differs from the broad-spectrum tissue expression patterns of *SAUR* genes in angiosperms such as Arabidopsis and cucumber [35,36], suggesting the functional specialization of conifers during long-term drought adaptation. It may represent a putative evolutionary feature developed by *P. massoniana* in response to seasonal drought conditions in southern regions.

It is noteworthy that the four core drought-responsive PmSAUR proteins (PmSAUR14/28/54/73) tend to be exclusively localized to the nucleus, which differs to some extent from canonical SAUR proteins that mainly function in the cytoplasm and plasma membrane [16]. Such nuclear localization may imply their potential specific regulatory capacity. These proteins could act as transcriptional regulators to integrate auxin signals, thereby indirectly participating in the modulation of plant drought tolerance [18]. This subcellular characteristic partly reflects the functional divergence of SAUR proteins between gymnosperms and herbaceous angiosperms, and also helps preliminarily elucidate the unique expression patterns and the potential mechanism underlying the reverse auxin regulation of *SAUR* genes in *P. massoniana*.

More importantly, this study identified that the key drought-responsive *SAUR* genes in *P. massoniana* exhibit an expression pattern consistent with an auxin-negative response, which differs from the conserved expression pattern where most classical *SAUR* genes are up-regulated by IAA induction [32,42]. This suggests a putative unique reverse regulatory mode characterized by drought-induced up-regulation and IAA-suppressed down-regulation. Under drought stress, the synthesis and polar transport of endogenous auxin in plants may be affected. It has been reported that IAA content decreases after drought stress [33,34]. Consequently, this reduction may lift the suppressive effect on such genes, potentially enabling their rapid upregulation. Among these genes, *PmSAUR54/73* targets root tissues, potentially contributing to enhancing water absorption and stress tolerance of *P. massoniana*, possibly through regulating root cell elongation and optimizing root architecture, as supported by their root-preferential expression (Figure 9) and drought-induced upregulation (Figure 8a) [35,36], while *PmSAUR14/28* specifically expressed in male cones, potentially helping to maintain the normal development and maintenance of reproductive function of reproductive organs under drought conditions. These two groups of genes may work synergistically to achieve a division of labor-drought-resistant roots underground and reproductive security aboveground-which could constitute a potential core molecular strategy for *P. massoniana* to balance growth and defense under drought stress. Simultaneously, four key genes displayed distinct gene-specific and temporal-specific expression patterns in response to MeJA [43]. *PmSAUR14* was rapidly induced at 3 h, while *PmSAUR28* showed delayed induction at 12 h. In contrast, the transcript levels of *PmSAUR54* and *PmSAUR73* were significantly down-regulated. This response pattern was highly correlated with the abundance distribution of MeJA-responsive elements in the promoter regions, suggesting that the *SAUR* genes of *P. massoniana* may integrate the dual hormonal signals of IAA and MeJA. This finding suggests the potential uniqueness of the *SAUR* family in woody conifers, which differs from herbaceous angiosperms in terms of hormone response patterns and functional differentiation, thereby potentially partially filling the research gap in the hormonal regulatory mechanisms of *SAUR* genes in gymnosperms.

Based on the expression data, we hypothesize that the auxin-negative-responsive *SAUR* genes (*PmSAUR14/28/54/73*) may participate in drought adaptation through tissue-specific regulation. Specifically, *PmSAUR54/73* are hypothesized to influence root system remodeling, while *PmSAUR14/28* may affect male cone development under drought stress. These hypotheses require functional validation in future studies.

We acknowledge that direct measurement of endogenous IAA under our specific experimental conditions and combined drought + IAA treatment were not performed. Therefore, the proposed inverse regulation pattern remains correlative and requires further experimental validation, including IAA quantification, combined treatments, and promoter functional assays.

## 5. Conclusions

Based on the chromosome-level genome of *P. massoniana*, this study identified 73 members of the *SAUR* gene family through genome-wide analysis and systematically elucidated the physicochemical properties, chromosomal localization, phylogenetic relationships, gene structures, and promoter cis-acting elements of *PmSAUR* genes. It was determined that tandem duplication serves as the primary driving force for the expansion of the *PmSAUR* family, which comprises both conserved branches homologous to angiosperms and unique branches specific to *P. massoniana*. Motif 1 was identified as the core essential motif, and the MeJA signaling pathway plays a central role in its regulation. Furthermore, through transcriptome sequencing and molecular experiments, it was discovered that 38 *PmSAUR* genes participate in the drought stress response and exhibit diverse temporal expression patterns. Four key drought-responsive genes *(PmSAUR14*, *PmSAUR28*, *PmSAUR54*, and *PmSAUR73*) localized in the nucleus were identified, which showed tissue-specific high expression in male cones or roots. These genes exhibit an expression pattern consistent with an auxin-negative response and display a unique regulatory pattern characterized by drought-induced upregulation and IAA-mediated downregulation. They mediate the coordinated drought adaptation strategy between aboveground reproductive organ regulation and underground root system adaptation in *P. massoniana*, serving as core molecular modules that balance growth and defense under drought stress. The above findings provide a new theoretical basis for elucidating the molecular regulatory network of drought stress response in conifers, enriching the evolutionary and functional research system of the plant *SAUR* gene family. Although *PmSAUR22* and *PmSAUR37* showed auxin-induced expression patterns, they are not the focus of the present study and are presented as contrasting examples.

## Data Availability

The original contributions presented in this study are included in the article and Supplementary Materials. Raw transcriptome reads have been deposited in the NCBI Sequence Read Archive (SRA) under BioProject accession PRJNA595650. The genome assembly version used in this study is *Pinus massoniana* v1.0. The nucleotide and protein sequences of *P. massoniana PmSAUR* genes are available in the GigaScience Database at https://gigadb.org/dataset/102688, (accessed on 16 October 2025).

## Supplementary Materials

The following supporting information can be downloaded at: https://www.mdpi.com/article/10.3390/biology15120962/s1, Figure S1: Distribution of cis-acting regulatory elements in the promoters of *Pinus massoniana SAUR* genes. Table S1: Characteristics and subcellular localization prediction of *PmSAUR*s; Table S2: qPCR Primers of *PmSAUR* Genes; Table S3: Adaptor Primers of *PmSAUR* Genes; Table S4. Effect sizes (Cohen’s d) and 95% confidence intervals for the *PmSAUR* gene.
