# Supplementary material for: Identification of the SAUR Gene Family in Pinus massoniana and Analysis of Its Expression Patterns Under Drought Stress
Source: Biology (Basel). 2026 Jun 19;15(12):962. doi: 10.3390/biology15120962 (PMC13295460; doi:10.3390/biology15120962)
Supplement: Supplementary file 1 [file biology-15-00962-s001.zip › Figure S1 caption.pdf]

Figure S1 Distribution of cis-acting regulatory elements in the promoters of Pinus massoniana SAUR genes. Each row represents a PmSAUR gene, with the gene name labeled on the left. The horizontal axis indicates the position on the 2000 bp promoter region upstream of the transcription start site (from 0 to 2000 bp). Colored dots mark the locations of different cis-acting regulatory elements, with their corresponding functions explained in the legend on the right.
