# Supplementary material for: Identification of the SAUR Gene Family in Pinus massoniana and Analysis of Its Expression Patterns Under Drought Stress
Source: Biology (Basel). 2026 Jun 19;15(12):962. doi: 10.3390/biology15120962 (PMC13295460; doi:10.3390/biology15120962)
Supplement: Supplementary file 1 [file biology-15-00962-s001.zip › Table S1.pdf]

Table S1 Sequence characteristics and subcellular localization prediction of members of the SAUR gene family of *Pinus massoniana*

Gene name	Gene ID	amino acids length/aa	Isoelectric point (PI)	Protein molecular mass/kDa	Instability index	GRAVY	Predicted subcellular localization
PmSAUR1	gmmutg101319G000010.1	169	9.17	19.27	39.98	-0.42	Mitochondrial
PmSAUR2	gmmutg10213G000010.1	113	9.47	12.86	43.17	0.02	Mitochondrial
PmSAUR3	gmmutg10213G000020.1	116	9.61	13.28	37.96	-0.18	Mitochondrial
PmSAUR4	gmmutg10213G000030.1	110	7.77	12.38	42.27	0.01	Nuclear
PmSAUR5	gmmutg11092G000010.1	138	7.77	15.89	39.96	-0.41	Mitochondrial
PmSAUR6	gmmutg115366G000020.1	146	9.22	16.81	53.41	0.06	PlasmaMembrane
PmSAUR7	gmmutg115366G000030.1	114	9.24	13.10	46.34	-0.09	Mitochondrial
PmSAUR8	gmmutg11611G000050.1	147	6.83	16.42	55.25	-0.23	Extracellular
PmSAUR9	gmmutg120094G000010.1	196	9.98	22.11	49.88	-0.14	Mitochondrial
PmSAUR10	gmmutg124804G000010.1	115	8.55	12.80	43.20	0.11	Extracellular
PmSAUR11	gmmutg13963G000050.1	155	9.69	18.26	52.37	-0.18	Mitochondrial
PmSAUR12	gmmutg13963G000070.1	175	9.44	20.45	55.11	-0.15	PlasmaMembrane
PmSAUR13	gmmutg140923G000010.1	135	9.56	16.07	67.64	-0.31	Nuclear
PmSAUR14	gmmutg140931G000010.1	118	7.84	14.01	49.73	-0.60	Nuclear
PmSAUR15	gmmutg140931G000050.1	118	7.17	13.97	52.48	-0.66	Nuclear
PmSAUR16	gmmutg16417G000060.1	132	9.17	14.91	29.43	-0.07	Mitochondrial
PmSAUR17	gmmutg165759G000010.1	118	7.83	13.46	43.53	0.01	Nuclear
PmSAUR18	gmmutg1714G000070.1	181	10.18	20.61	41.96	-0.16	Mitochondrial
PmSAUR19	gmmutg18444G000020.1	120	9.26	13.58	32.5	-0.05	Mitochondrial
PmSAUR20	gmmutg20280G000080.1	193	8.79	21.26	50.15	-0.19	Mitochondrial
PmSAUR21	gmmutg2125G000010.1	150	9.14	17.28	47.93	-0.31	Mitochondrial
PmSAUR22	gmmutg2125G000040.1	149	9.41	17.11	40.74	-0.23	Mitochondrial
PmSAUR23	gmmutg2125G000050.1	107	9.57	12.52	52.52	-0.30	Mitochondrial
PmSAUR24	gmmutg2125G000060.1	152	8.19	17.21	40.77	-0.16	Mitochondrial
PmSAUR25	gmmutg2125G000070.1	152	9.15	17.26	45.17	-0.14	Mitochondrial
PmSAUR26	gmmutg223610G000010.1	144	7.69	16.48	28.62	-0.33	Mitochondrial
PmSAUR27	gmmutg227652G000010.1	196	9.18	21.83	36.3	-0.04	Mitochondrial
PmSAUR28	gmmutg23040G000020.1	118	7.83	13.98	47.14	-0.56	Nuclear
PmSAUR29	gmmutg25572G000040.1	126	8.64	14.62	42.62	-0.18	Mitochondrial
PmSAUR30	gmmutg25618G000020.1	153	9.54	17.36	41.08	-0.18	Mitochondrial
PmSAUR31	gmmutg25882G000010.1	155	7.69	17.60	37.74	-0.24	Mitochondrial
PmSAUR32	gmmutg260564G000010.1	132	9.48	15.24	58.28	-0.25	Nuclear
PmSAUR33	gmmutg26266G000040.1	134	8.97	15.50	43.47	0.08	Mitochondrial
PmSAUR34	gmmutg27325G000010.1	150	6.59	17.10	58.7	-0.04	Cytoplasmic
PmSAUR35	gmmutg287081G000010.1	196	9.63	21.87	40.58	-0.07	Mitochondrial
PmSAUR36	gmmutg293435G000010.1	118	9	13.54	46.55	-0.04	Nuclear
PmSAUR37	gmmutg3322G000020.1	143	9.34	16.57	58.99	-0.51	Extracellular
PmSAUR38	gmmutg3353G000010.1	115	7.84	13.25	58.89	-0.05	Nuclear

PmSAUR39	gmmutg3353G000020.1	118	8.77	13.38	40.29	-0.10	Nuclear
PmSAUR40	gmmutg34521G000020.1	149	6.04	16.62	40.43	-0.06	Cytoplasmic
PmSAUR41	gmmutg37897G000010.1	114	9.77	13.03	74.66	-0.09	Mitochondrial
PmSAUR42	gmmutg37897G000040.1	114	8.99	12.89	69.43	-0.08	Nuclear
PmSAUR43	gmmutg4011G000030.1	167	9.26	18.85	36.51	-0.25	Mitochondrial
PmSAUR44	gmmutg42016G000020.1	196	9.54	21.74	39.71	-0.11	Mitochondrial
PmSAUR45	gmmutg43021G000010.1	194	9.24	21.71	40.83	-0.29	Mitochondrial
PmSAUR46	gmmutg47369G000010.1	137	5.48	15.46	39.17	-0.47	Nuclear
PmSAUR47	gmmutg47800G000020.1	113	8.5	12.70	33.3	0.03	Mitochondrial
PmSAUR48	gmmutg49266G000010.1	118	9.93	13.55	44.02	-0.22	Mitochondrial
PmSAUR49	gmmutg49266G000030.1	118	8.59	13.53	42.64	-0.18	Mitochondrial
PmSAUR50	gmmutg5124G000130.1	170	10.31	20.21	40.86	-0.41	Mitochondrial
PmSAUR51	gmmutg5124G000140.1	170	10.31	20.16	38.72	-0.39	Mitochondrial
PmSAUR52	gmmutg5401G000030.1	131	8.44	14.91	52.27	-0.58	Nuclear
PmSAUR53	gmmutg5401G000040.1	158	6.96	18.03	59.23	-0.45	Nuclear
PmSAUR54	gmmutg6072G000020.1	177	9.4	20.05	55.02	-0.41	Nuclear
PmSAUR55	gmmutg611G000060.1	177	6.65	20.01	51.16	-0.23	Mitochondrial
PmSAUR56	gmmutg6344G000030.1	196	10.02	22.19	40.57	-0.17	Mitochondrial
PmSAUR57	gmmutg6800G000020.1	117	6.29	13.52	53.74	-0.23	Mitochondrial
PmSAUR58	gmmutg68867G000010.1	186	9.54	20.61	36.78	-0.13	Nuclear
PmSAUR59	gmmutg709G000070.1	201	10.34	23.34	48.21	-0.55	Nuclear
PmSAUR60	gmmutg7120G000020.1	155	8.5	17.85	61.18	-0.44	Nuclear
PmSAUR61	gmmutg7120G000030.1	177	8.32	19.75	52.38	-0.32	Mitochondrial
PmSAUR62	gmmutg72781G000010.1	114	9.15	12.95	46.97	-0.04	Mitochondrial
PmSAUR63	gmmutg72781G000020.1	113	9.72	12.78	38.6	0.05	Mitochondrial
PmSAUR64	gmmutg72781G000030.1	114	8.89	12.92	36.41	0.03	Mitochondrial
PmSAUR65	gmmutg72781G000040.1	118	9.74	13.54	48.54	-0.01	Mitochondrial
PmSAUR66	gmmutg7362G000030.1	175	9.91	20.07	48.24	-0.21	Mitochondrial
PmSAUR67	gmmutg7438G000010.1	149	8.78	16.97	69.19	-0.23	Nuclear
PmSAUR68	gmmutg75619G000010.1	118	9.14	13.74	57.35	-0.31	Nuclear
PmSAUR69	gmmutg78695G000010.1	152	9.23	17.45	55.94	-0.23	Mitochondrial
PmSAUR70	gmmutg82553G000040.1	152	9.42	17.54	56.1	-0.25	Mitochondrial
PmSAUR71	gmmutg9117G000160.1	161	9.33	18.21	53.11	-0.42	Mitochondrial
PmSAUR72	gmmutg91879G000020.1	125	8.49	14.26	48.29	-0.09	Nuclear
PmSAUR73	MSTRG.16480.6.p1	258	9.08	29.28	69.38	-0.43	Nuclear
