# Supplementary material for: Identification of the SAUR Gene Family in Pinus massoniana and Analysis of Its Expression Patterns Under Drought Stress
Source: Biology (Basel). 2026 Jun 19;15(12):962. doi: 10.3390/biology15120962 (PMC13295460; doi:10.3390/biology15120962)
Supplement: Supplementary file 1 [file biology-15-00962-s001.zip › Table S2.pdf]

Table S2 qPCR Primers of *PmSAUR* Genes

Gene name	Primer Names	Sequence (5′-3′)
PmSAUR14	F	AAACTGCTGAAGGAAGCCGA
	R	GCTGTGAGAGTGGTGGTGTT
PmSAUR28	F	ACAACCTCAGCGTTTCGTGA
	R	GAAGTCCGAGACCTGGCAAG
PmSAUR54	F	GAGGCGGTTCATCATTCCCA
	R	GGCCTCCCTGATGATCGAAG
PmSAUR73	F	AGGGGCAGGTTCAATGTCTG
	R	CCCTATACGGCGTGACGAAA
PmSAUR22	F	GCGGTGTATGTTGGGAAGGA
	R	ACTCCTCTTCGGCCTTCTCT
PmSAUR37	F	CCGAAAGCTGCAGAGAGTGA
	R	GGGACATCGGTGTAACAGCA
