# Supplementary material for: Identification of the SAUR Gene Family in Pinus massoniana and Analysis of Its Expression Patterns Under Drought Stress
Source: Biology (Basel). 2026 Jun 19;15(12):962. doi: 10.3390/biology15120962 (PMC13295460; doi:10.3390/biology15120962)
Supplement: Supplementary file 1 [file biology-15-00962-s001.zip › Table S3.pdf]

Table S3 Adaptor Primers of *PmSAUR* Genes

Gene name	Primer Names	Sequence (5′-3′)
PmSAUR14	F	ctcttgaccatggATGCCTCATTCAC
	R	agtcagatctCCGAAAACAGAAGGTA
PmSAUR28	F	ctcttgaccatggATGCCCCATTCACTGCA
	R	gtcagatctCCGAAAACAGAAGGTAG
PmSAUR54	F	cttgaccatggATGAAGGGCGAGAAA
	R	agtcagatctGCCGAACGATTTCTGG
PmSAUR73	F	cttgaccatggCTACCACGCCATGTTCAT
	R	agtcagatctCCTAGTGCTGTCTTGC
