# Supplementary material for: Identification of the SAUR Gene Family in Pinus massoniana and Analysis of Its Expression Patterns Under Drought Stress
Source: Biology (Basel). 2026 Jun 19;15(12):962. doi: 10.3390/biology15120962 (PMC13295460; doi:10.3390/biology15120962)
Supplement: Supplementary file 1 [file biology-15-00962-s001.zip › Table S4.pdf]

Table S4. Effect sizes (Cohen's d) and 95% confidence intervals for the *PmSAUR* gene

Table S4.1. Effect sizes (Cohen's d) and 95% confidence intervals for the PmSAUR genes under different drought treatments

Gene name	Comparison group	Cohen's d	lower 95% CI	upper 95% CI
	Drought 0d vs Drought 3d	0.655	-0.197	1.453
	Drought 0d vs Drought 7d	0.699	-0.169	1.513
	Drought 0d vs Drought 12d	3.505	1.303	5.706
	Drought 0d vs Drought 20d	2.435	0.817	4.029
	Drought 0d vs RW 3d	1.586	0.299	2.831
	Drought 3d vs Drought 7d	2.458	0.488	4.447
PmSAUR14	Drought 3d vs Drought 12d	0.632	-0.212	1.422
	Drought 3d vs Drought 20d	0.658	-0.195	1.457
	Drought 3d vs RW 3d	0.646	-0.249	1.477
	Drought 7d vs Drought 12d	0.661	-0.194	1.461
	Drought 12d vs Drought 20d	1.847	0.533	3.126
	Drought 12d vs RW 3d	2.078	0.519	3.612
	Drought 20d vs RW 3d	1.133	0.077	2.136
	Drought 0d vs Drought 3d	1.639	0.199	3.052
	Drought 0d vs Drought 7d	5.194	1.309	9.221
	Drought 0d vs Drought 12d	2.543	0.516	4.594
	Drought 0d vs Drought 20d	3.353	1.034	5.688
	Drought 0d vs RW 3d	1.511	0.149	2.839
	Drought 3d vs Drought 20d	3.174	0.964	5.394
PmSAUR22	Drought 3d vs RW 3d	5.547	1.409	9.84
	Drought 7d vs Drought 12d	3.429	0.794	6.132
	Drought 7d vs Drought 20d	4.676	1.53	7.871
	Drought 7d vs RW 3d	1.802	0.26	3.327
	Drought 12d vs Drought 20d	5.813	1.945	9.756
	Drought 12d vs RW 3d	2.942	0.643	5.284
	Drought 20d vs RW 3d	2.857	0.84	4.875
	Drought 0d vs Drought 12d	8.186	2.142	14.484
	Drought 0d vs Drought 20d	3.61	0.849	6.448
	Drought 0d vs RW 3d	4.206	1.356	7.093
	Drought 3d vs Drought 7d	6.935	1.797	12.282
	Drought 3d vs Drought 12d	1.485	0.139	2.795
PmSAUR28	Drought 3d vs Drought 20d	1.249	0.042	2.408
	Drought 3d vs RW 3d	1.404	0.213	2.548
	Drought 7d vs Drought 12d	1.387	0.1	2.633
	Drought 7d vs Drought 20d	1.186	0.015	2.306
	Drought 7d vs RW 3d	1.315	0.169	2.411
	Drought 20d vs RW 3d	3.7	1.166	6.259
	Drought 0d vs Drought 3d	6.439	2.17	10.795
PmSAUR37	Drought 0d vs Drought 7d	1.262	0.143	2.331
	Drought 0d vs Drought 12d	2.095	0.527	3.639

	Drought 0d vs Drought 20d	1.903	0.443	3.332
	Drought 0d vs RW 3d	6.204	2.085	10.405
	Drought 3d vs Drought 7d	1.128	-0.01	2.212
	Drought 3d vs Drought 12d	2.056	0.351	3.758
	Drought 7d vs Drought 12d	0.858	-0.137	1.791
	Drought 7d vs Drought 20d	0.831	-0.151	1.749
	Drought 7d vs RW 3d	1.298	0.063	2.488
	Drought 12d vs Drought 20d	4.11	0.997	7.321
	Drought 12d vs RW 3d	1.575	0.174	2.945
	Drought 20d vs RW 3d	1.43	0.117	2.704
	Drought 0d vs Drought 3d	2.34	0.448	4.244
	Drought 0d vs Drought 7d	1.63	0.195	3.037
	Drought 0d vs Drought 12d	1.945	0.312	3.569
	Drought 0d vs Drought 20d	3.58	0.839	6.395
	Drought 0d vs RW 3d	1.714	0.227	3.179
	Drought 3d vs Drought 7d	4.026	0.972	7.174
	Drought 3d vs Drought 12d	7.37	1.918	13.047
PmSAUR54	Drought 3d vs Drought 20d	5.97	1.528	10.585
	Drought 3d vs RW 3d	5.465	1.386	9.696
	Drought 7d vs Drought 12d	4.877	1.219	8.664
	Drought 7d vs Drought 20d	2.658	0.553	4.792
	Drought 7d vs RW 3d	6.446	1.661	11.421
	Drought 12d vs Drought 20d	3.799	0.905	6.777
	Drought 12d vs RW 3d	7.944	2.076	14.058
	Drought 20d vs RW 3d	3.017	0.667	5.415
	Drought 0d vs Drought 3d	2.725	0.575	4.908
	Drought 0d vs Drought 7d	3.127	0.701	5.606
	Drought 0d vs Drought 12d	2.568	0.524	4.638
	Drought 0d vs Drought 20d	3.626	1.138	6.137
	Drought 0d vs RW 3d	2.492	0.499	4.507
	Drought 3d vs Drought 7d	5.084	1.278	9.028
	Drought 3d vs Drought 12d	4.744	1.181	8.431
PmSAUR73	Drought 3d vs Drought 20d	6.876	2.326	11.522
	Drought 3d vs RW 3d	7.482	1.948	13.243
	Drought 7d vs Drought 12d	4.281	1.047	7.62
	Drought 7d vs Drought 20d	6.735	2.276	11.287
	Drought 7d vs RW 3d	5.344	1.352	9.484
	Drought 12d vs Drought 20d	5.692	1.901	9.555
	Drought 12d vs RW 3d	5.497	1.395	9.753
	Drought 20d vs RW 3d	7.117	2.413	11.923

Table S4.2. Effect sizes (Cohen's d) and 95% confidence intervals for PmSAUR genes under different IAA treatments

Gene name	Comparison group	Cohen's d	lower 95% CI	upper 95% CI
PmSAUR14	IAA 0h vs IAA 3h	2.867	1.017	4.704
	IAA 0h vs IAA 6h	3.712	1.171	6.279
	IAA 0h vs IAA 12h	2.201	0.572	3.811
	IAA 0h vs IAA 24h	1.636	0.323	2.91
	IAA 3h vs IAA 6h	4.025	1.289	6.794
	IAA 3h vs IAA 12h	2.991	0.893	5.095
	IAA 3h vs IAA 24h	2.021	0.495	3.522
	IAA 6h vs IAA 12h	3.098	0.692	5.556
	IAA 6h vs IAA 24h	1.765	0.246	3.264
	IAA 12h vs IAA 24h	2.095	0.365	3.825
PmSAUR22	IAA 0h vs IAA 3h	0.899	-0.051	1.793
	IAA 0h vs IAA 6h	0.932	-0.032	1.84
	IAA 0h vs IAA 12h	1.106	0.062	2.095
	IAA 0h vs IAA 24h	2.664	0.763	4.561
	IAA 3h vs IAA 6h	11.762	3.118	20.787
	IAA 3h vs IAA 12h	3.32	0.761	5.942
	IAA 3h vs IAA 24h	1.519	0.153	2.852
	IAA 6h vs IAA 12h	3.32	0.761	5.942
	IAA 6h vs IAA 24h	1.184	0.014	2.303
	IAA 12h vs IAA 24h	1.519	0.153	2.852
PmSAUR28	IAA 0h vs IAA 3h	1.868	0.543	3.157
	IAA 0h vs IAA 6h	1.956	0.587	3.291
	IAA 0h vs IAA 12h	1.258	0.222	2.246
	IAA 0h vs IAA 24h	1.265	0.225	2.256
	IAA 3h vs IAA 6h	2.318	0.762	3.848
	IAA 3h vs IAA 12h	2.22	0.716	3.697
	IAA 3h vs IAA 24h	2.532	0.863	4.181
	IAA 6h vs IAA 12h	1.411	0.306	2.47
	IAA 6h vs IAA 24h	1.461	0.333	2.544
	IAA 12h vs IAA 24h	2.122	0.668	3.546
PmSAUR37	IAA 0h vs IAA 3h	2.51	0.7	4.31
	IAA 0h vs IAA 6h	2.309	0.617	3.984
	IAA 0h vs IAA 12h	3.778	1.423	6.138
	IAA 0h vs IAA 24h	2.913	0.862	4.966

	IAA 3h vs IAA 6h	1.253	0.044	2.413
	IAA 3h vs IAA 12h	2.137	0.544	3.706
	IAA 3h vs IAA 24h	3.074	0.685	5.514
	IAA 6h vs IAA 12h	2.007	0.489	3.498
	IAA 6h vs IAA 24h	1.297	0.063	2.486
	IAA 12h vs IAA 24h	2.388	0.65	4.111
PmSAUR54	IAA 0h vs IAA 3h	3.661	0.864	6.536
	IAA 0h vs IAA 6h	2.862	0.618	5.146
	IAA 0h vs IAA 12h	2.774	0.59	4.993
	IAA 0h vs IAA 24h	0.966	-0.085	1.956
	IAA 3h vs IAA 6h	1.771	0.248	3.274
	IAA 3h vs IAA 12h	1.782	0.253	3.293
	IAA 3h vs IAA 24h	0.853	-0.14	1.783
	IAA 6h vs IAA 12h	3.906	0.937	6.965
	IAA 6h vs IAA 24h	1.178	0.012	2.293
	IAA 12h vs IAA 24h	1.107	-0.02	2.179
PmSAUR73	IAA 0h vs IAA 3h	4.221	1.029	7.515
	IAA 0h vs IAA 6h	1.198	0.11	2.233
	IAA 0h vs IAA 12h	1.167	0.094	2.186
	IAA 0h vs IAA 24h	1.144	0.082	2.153
	IAA 3h vs IAA 6h	1.524	0.27	2.734
	IAA 3h vs IAA 12h	1.491	0.255	2.682
	IAA 3h vs IAA 24h	1.446	0.233	2.614
	IAA 6h vs IAA 12h	3.237	1.184	5.284
	IAA 6h vs IAA 24h	2.995	1.075	4.904
	IAA 12h vs IAA 24h	4.355	1.673	7.05

Table S4.3. Effect sizes (Cohen's d) and 95% confidence intervals for PmSAUR genes under different methyl jasmonate (MeJA) treatments

Gene name	Comparison group	Cohen's d	lower 95% CI	upper 95% CI
PmSAUR14	MeJA 0h vs MeJA 3h	1.87	0.285	3.441

	MeJA 0h vs MeJA 6h	1.87	0.285	3.441
	MeJA 0h vs MeJA 12h	3.384	0.78	6.054
	MeJA 0h vs MeJA 24h	3.027	0.67	5.432
	MeJA 3h vs MeJA 6h	2.949	0.646	5.296
	MeJA 3h vs MeJA 12h	3.403	0.786	6.086
	MeJA 3h vs MeJA 24h	1.26	0.047	2.426
	MeJA 6h vs MeJA 12h	7.442	1.937	13.174
	MeJA 6h vs MeJA 24h	1.893	0.293	3.481
	MeJA 12h vs MeJA 24h	1.736	0.236	3.216
	MeJA 0h vs MeJA 3h	2.454	0.486	4.44
	MeJA 0h vs MeJA 6h	1.168	0.007	2.277
	MeJA 0h vs MeJA 12h	0.979	-0.079	1.976
	MeJA 0h vs MeJA 24h	1.698	0.221	3.151
PmSAUR22	MeJA 3h vs MeJA 6h	1.859	0.281	3.423
	MeJA 3h vs MeJA 12h	1.339	0.08	2.555
	MeJA 3h vs MeJA 24h	4.046	0.978	7.21
	MeJA 6h vs MeJA 12h	3.513	0.819	6.278
	MeJA 6h vs MeJA 24h	2.615	0.539	4.719
	MeJA 12h vs MeJA 24h	1.668	0.21	3.101
	MeJA 0h vs MeJA 3h	3.324	1.023	5.641
	MeJA 0h vs MeJA 6h	6.257	2.104	10.492
	MeJA 0h vs MeJA 12h	0.864	-0.072	1.741
	MeJA 0h vs MeJA 24h	2.985	0.89	5.084
PmSAUR28	MeJA 3h vs MeJA 6h	3.194	0.722	5.722
	MeJA 3h vs MeJA 12h	0.993	-0.072	1.999
	MeJA 3h vs MeJA 24h	1.591	0.181	2.973
	MeJA 6h vs MeJA 12h	0.865	-0.134	1.801
	MeJA 6h vs MeJA 24h	2.646	0.549	4.772
	MeJA 12h vs MeJA 24h	0.762	-0.186	1.645
	MeJA 0h vs MeJA 3h	5.832	1.49	10.342
	MeJA 0h vs MeJA 6h	1.984	0.326	3.635
	MeJA 0h vs MeJA 12h	1.355	0.087	2.581
PmSAUR37	MeJA 0h vs MeJA 24h	2.432	0.479	4.402
	MeJA 3h vs MeJA 6h	2.771	0.589	4.988
	MeJA 3h vs MeJA 12h	1.639	0.199	3.052
	MeJA 3h vs MeJA 24h	3.775	0.898	6.736
	MeJA 6h vs MeJA 12h	3.477	0.808	6.215

	MeJA 6h vs MeJA 24h	9.031	2.374	15.972
	MeJA 12h vs MeJA 24h	2.653	0.551	4.783
PmSAUR54	MeJA 0h vs MeJA 3h	2.323	0.765	3.857
	MeJA 0h vs MeJA 6h	3.507	1.093	5.941
	MeJA 0h vs MeJA 12h	4.316	1.397	7.275
	MeJA 0h vs MeJA 24h	4.403	1.429	7.418
	MeJA 3h vs MeJA 6h	3.657	1.15	6.187
	MeJA 3h vs MeJA 12h	3.672	1.156	6.212
	MeJA 3h vs MeJA 24h	3.63	1.139	6.142
	MeJA 6h vs MeJA 12h	6.364	1.638	11.276
	MeJA 6h vs MeJA 24h	6.355	1.636	11.261
	MeJA 12h vs MeJA 24h	25.455	6.81	44.945
PmSAUR73	MeJA 0h vs MeJA 3h	2.517	0.856	4.157
	MeJA 0h vs MeJA 6h	1.17	0.172	2.119
	MeJA 0h vs MeJA 12h	2.839	1.004	4.66
	MeJA 0h vs MeJA 24h	0.887	0.003	1.721
	MeJA 3h vs MeJA 6h	0.89	0.005	1.725
	MeJA 3h vs MeJA 12h	3.677	1.378	5.977
	MeJA 3h vs MeJA 24h	0.709	-0.111	1.483
	MeJA 6h vs MeJA 12h	0.954	0.044	1.813
	MeJA 6h vs MeJA 24h	0.819	-0.04	1.628
	MeJA 12h vs MeJA 24h	0.75	-0.085	1.536

Table S4.4. Effect sizes (Cohen's d) and 95% confidence intervals for the *PmSAUR* gene in different tissues

Gene name	Comparison group	Cohen's d	lower 95% CI	upper 95% CI
PmSAUR14	F vs FF	4.04	1.233	6.862
	F vs MF	0.738	-0.3	1.712
	F vs R	1.148	0.06	2.175
	F vs L	1.183	-0.032	2.327
	F vs YS	2.356	0.543	4.131
	F vs OS	0.918	-0.187	1.953
	FF vs MF	0.877	-0.353	2.019
	FF vs R	1.437	0.105	2.702
	FF vs L	1.527	-0.029	3.007

	FF vs YS	3.773	0.796	6.796
	MF vs L	0.918	-0.33	2.077
	MF vs YS	0.883	-0.349	2.028
	MF vs OS	0.972	-0.301	2.155
	R vs L	3.536	1.035	6.038
	R vs YS	1.627	0.201	2.989
	R vs OS	5.907	1.935	9.942
	L vs YS	1.969	0.159	3.726
	L vs OS	2.479	0.355	4.578
	YS vs OS	1.263	-0.152	2.592
	F vs MF	1.351	0.273	2.382
	F vs R	1.421	0.311	2.485
	F vs L	0.6	-0.233	1.38
	F vs YS	2.252	0.731	3.746
	F vs OS	1.027	0.088	1.916
	FF vs MF	1.217	0.199	2.187
	FF vs R	1.243	0.213	2.225
	FF vs L	0.597	-0.234	1.377
	FF vs YS	1.789	0.503	3.037
PmSAUR22	FF vs OS	0.976	0.058	1.844
	MF vs YS	1.593	0.403	2.742
	MF vs OS	1.772	0.495	3.012
	R vs L	0.596	-0.235	1.375
	R vs YS	1.271	0.229	2.265
	R vs OS	0.933	0.031	1.784
	L vs YS	0.606	-0.229	1.388
	L vs OS	0.677	-0.183	1.483
	YS vs OS	1.149	0.16	2.089
	F vs R	0.786	-0.117	1.632
	F vs L	0.853	-0.078	1.726
	F vs YS	0.97	-0.011	1.895
	F vs OS	1.144	0.082	2.152
	FF vs MF	0.829	-0.152	1.747
	FF vs R	3.083	0.688	5.529
PmSAUR28	FF vs L	4.03	0.973	7.182
	FF vs YS	2.779	0.592	5.002
	FF vs OS	1.855	0.279	3.417
	MF vs R	0.906	-0.113	1.864
	MF vs OS	0.736	-0.2	1.608
	R vs L	4.667	1.159	8.297
	R vs YS	2.262	0.422	4.111

	R vs OS	1.559	0.168	2.918
	L vs YS	3.749	0.89	6.69
	L vs OS	2.098	0.366	3.83
	YS vs OS	3.666	0.865	6.544
PmSAUR37	F vs FF	3.657	1.15	6.188
	F vs MF	0.68	-0.182	1.487
	F vs R	0.687	-0.177	1.497
	F vs L	1.29	0.239	2.292
	F vs YS	5.89	2.325	9.491
	F vs OS	1.741	0.371	3.076
	FF vs MF	0.745	-0.195	1.621
	FF vs R	0.755	-0.19	1.635
	FF vs L	0.938	-0.029	1.849
	FF vs YS	3.445	1.069	5.84
	FF vs OS	1.149	-0.001	2.246
	MF vs R	15.333	4.084	27.084
	MF vs L	0.608	-0.227	1.39
	MF vs YS	0.674	-0.185	1.478
	MF vs OS	0.66	-0.241	1.497
	R vs L	0.61	-0.226	1.393
	R vs YS	0.681	-0.181	1.488
	R vs OS	0.662	-0.24	1.501
	L vs YS	1.37	0.284	2.41
	L vs OS	2.556	0.719	4.384
	YS vs OS	1.917	0.449	3.355
PmSAUR54	F vs FF	1.227	0.204	2.202
	F vs MF	1.143	0.157	2.081
	F vs R	0.616	-0.222	1.402
	F vs L	1.281	0.152	2.36
	F vs YS	0.789	-0.115	1.636
	F vs OS	1.023	0.018	1.972
	FF vs MF	1.695	0.456	2.895
	FF vs R	0.657	-0.196	1.456
	FF vs L	1.83	0.411	3.215
	FF vs YS	1.077	0.046	2.051
	FF vs OS	1.584	0.299	2.828
	MF vs R	0.745	-0.142	1.575
	MF vs L	2.419	0.663	4.162
	MF vs YS	2.209	0.575	3.824
	MF vs OS	3.209	0.978	5.451
	R vs L	0.709	-0.214	1.568

	R vs YS	0.893	-0.12	1.843
	R vs OS	0.742	-0.197	1.617
	L vs YS	1.259	0.046	2.424
	L vs OS	2.361	0.455	4.281
	YS vs OS	1.645	0.201	3.062
	F vs FF	2.11	0.59	3.585
	F vs MF	1.581	0.311	2.795
	F vs R	0.759	-0.287	1.739
	F vs L	0.782	-0.272	1.77
	F vs YS	2.726	0.892	4.529
	F vs OS	1.322	0.164	2.421
	FF vs MF	2.408	0.738	4.039
	FF vs R	0.785	-0.27	1.773
	FF vs L	0.829	-0.242	1.832
	FF vs YS	3.084	1.06	5.085
PmSAUR73	FF vs OS	1.028	-0.014	2.01
	MF vs R	0.815	-0.251	1.813
	MF vs L	0.884	-0.208	1.907
	MF vs YS	2.267	0.669	3.824
	MF vs OS	0.965	-0.053	1.925
	R vs L	3.124	0.583	5.678
	R vs YS	0.778	-0.275	1.764
	R vs OS	0.736	-0.302	1.708
	L vs YS	0.816	-0.25	1.815
	L vs OS	0.741	-0.299	1.715
	YS vs OS	1.116	0.041	2.131
